# Mode Equivalence of Health Indicators Between Data Collection Modes and Mixed-Mode Survey Designs in Population-Based Health Interview Surveys for Children and Adolescents: Methodological Study

**DOI:** 10.2196/jmir.7802

**Published:** 2018-03-05

**Authors:** Elvira Mauz, Robert Hoffmann, Robin Houben, Laura Krause, Panagiotis Kamtsiuris, Antje Gößwald

**Affiliations:** ^1^ Department of Epidemiology and Health Monitoring Robert Koch Institute Berlin Germany

**Keywords:** public health, child and adolescent health, health surveys, survey methods, mixed-mode survey, paper-and-pencil questionnaire, online questionnaire, mode effects

## Abstract

**Background:**

The implementation of an Internet option in an existing public health interview survey using a mixed-mode design is attractive because of lower costs and faster data availability. Additionally, mixed-mode surveys can increase response rates and improve sample composition. However, mixed-mode designs can increase the risk of measurement error (mode effects).

**Objective:**

This study aimed to determine whether the prevalence rates or mean values of self- and parent-reported health indicators for children and adolescents aged 0-17 years differ between self-administered paper-based questionnaires (SAQ-paper) and self-administered Web-based questionnaires (SAQ-Web), as well as between a single-mode control group and different mixed-mode groups.

**Methods:**

Data were collected for a methodological pilot of the third wave of the "German Health Interview and Examination Survey for Children and Adolescents". Questionnaires were completed by parents or adolescents. A population-based sample of 11,140 children and adolescents aged 0-17 years was randomly allocated to 4 survey designs—a single-mode control group with paper-and-pencil questionnaires only (n=970 parents, n=343 adolescents)—and 3 mixed-mode designs, all of which offered Web-based questionnaire options. In the concurrent mixed-mode design, both questionnaires were offered at the same time (n=946 parents, n=290 adolescents); in the sequential mixed-mode design, the SAQ-Web was sent first, followed by the paper questionnaire along with a reminder (n=854 parents, n=269 adolescents); and in the preselect mixed-mode design, both options were offered and the respondents were asked to request the desired type of questionnaire (n=698 parents, n=292 adolescents). In total, 3468 questionnaires of parents of children aged 0-17 years (SAQ-Web: n=708; SAQ-paper: n=2760) and 1194 questionnaires of adolescents aged 11-17 years (SAQ-Web: n=299; SAQ-paper: n=895) were analyzed. Sociodemographic characteristics and a broad range of health indicators for children and adolescents were compared by survey design and data collection mode by calculating predictive margins from regression models.

**Results:**

There were no statistically significant differences in sociodemographic characteristics or health indicators between the single-mode control group and any of the mixed-mode survey designs. Differences in sociodemographic characteristics between SAQ-Web and SAQ-paper were found. Web respondents were more likely to be male, have higher levels of education, and higher household income compared with paper respondents. After adjusting for sociodemographic characteristics, only one of the 38 analyzed health indicators showed different prevalence rates between the data collection modes, with a higher prevalence rate for lifetime alcohol consumption among the online-responding adolescents (*P*<.001).

**Conclusions:**

These results suggest that mode bias is limited in health interview surveys for children and adolescents using a mixed-mode design with Web-based and paper questionnaires.

## Introduction

The assessment of population health using health interview surveys is an established method in many countries and is a cornerstone of health reporting, health policies, and health sciences. However, epidemiological studies have shown decreasing response rates since the 1990s [1-3]. The use of mixed-mode health interview surveys offers respondents various data collection modes and can increase the response rate, improve sample composition, and reduce overall costs [3,4]. Currently, there is considerable interest in using Web-based health survey interviews because of lower costs and faster data availability. Web-based surveys are increasingly becoming standard [5], and they are frequently combined with other modes in mixed-mode designs [6]. However, the use of different survey modes may increase the risk of measurement error (mode effects) [5].

Mode effects are systematic distortions caused by different survey modes or interview situations [5]. They often arise when there are large methodological differences in the survey situation (self-administered questionnaire vs interviews) or the communication channel (auditory vs visual) [3]. Such differences are minimal between self-administered paper-based questionnaires (SAQ-paper) and self-administered Web-based questionnaires (SAQ-Web)—both are conducted without an interviewer and both use visual perception. For this reason, these 2 self-administered modes (SAQ-Web and SAQ-paper) are considered mode equivalent [4,7,8]. Mode equivalence is shown if an individual gives the same response to the same question or instrument administered through 2 different modes, leading to the same results [9]. For example, research has shown no differences between the 2 data collection modes in prevalence rates of diseases among adult populations [10,11] or in reported health behaviors among adolescents [12].

However, researchers have discussed mode effects for sensitive topics. Web-based responses are associated with both anonymity and greater individualization. Consequently, SAQ-Web participants are not affected by social desirability; rather, they are less orientated toward social norms. Therefore, SAQ-Web mode yields the most honest reports, especially compared with interview modes [13,14]. Furthermore, differences have been found between the 2 self-administered modes, for example, in political attitudes [15], reporting of sensitive sexual behaviors [16], or adolescent risk behavior [17]. However, there is high consistency of responses across modes, with only a few respondents taking advantage of the greater privacy of the Web mode [16]. Hence, possible mode effects should be investigated before changing or adding modes to existing health surveys. In ongoing longitudinal studies, changing the mode or offering a second mode may risk time-based comparability.

The German Health Interview and Examination Survey for Children and Adolescents (KiGGS) is a nationally representative health interview and examination survey of children and adolescents in Germany [18,19]. It is part of the nationwide health monitoring system administered by the German national public health institute (Robert Koch Institute) [20,21]. KiGGS obtains representative cross-sectional information on German children and adolescents aged 0-17 years at regular intervals. Additionally, based on the first cross-sectional sample (KiGGS baseline; 2003-2006), a KiGGS cohort has been implemented. The baseline respondents are being followed throughout their life course into adulthood [21]. The survey involves physical examinations and tests, as well as laboratory analysis of urine and blood parameters. All the parents and adolescents aged 1117 years completed paper-based questionnaires [20]. The first follow-up, KiGGS Wave 1 (2009-2012), was conducted using telephone interviews of parents and adolescents [22]. KiGGS Wave 2 (2014-2017) involved a health interview and examination, continuing the baseline concept [23]. The aim of the KiGGS survey is to provide current data on population health, health determinants, and the utilization of health care services. In addition, information is gathered about the incidence of disorders as well as trajectories of multiple health indicators throughout the life course. The data are widely used in national health reporting, health policies, and public health research.

When planning population-based (health) studies like KiGGS, the survey design must minimize total survey error [24,25]. In addition to lower data quality owing to measurement errors such as mode effects, the total survey error comprises different kinds of systematic errors—an undervalued sample size leads to imprecise estimates (sampling error) and the composition of the sample might be different from the target population (coverage error) owing to errors in the sampling procedure or because of systematic nonresponse (nonresponse bias). All these aspects were examined in a methodological pilot study as part of the KiGGS Wave 2 pretest. The pilot study aimed to compare 3 mixed-mode survey designs using Web- and paper-based questionnaires with a single-mode SAQ-paper design in terms of response rates, sample composition, data quality, and effort [26]. The study also explored whether estimates of health indicators differed among the survey designs and data collection modes. This study focused only on the second aim of the pilot study and addressed 2 research questions:

- Are there any differences in the prevalence rates or mean values of core public health indicators for children and adolescents aged 0-17 years between the single-mode control group using only SAQ-paper and different mixed-mode groups that combine offers of SAQ-paper and SAQ-Web?
- Are there any differences in prevalence rates or mean values of these indicators between the 2 data collection modes (SAQ-paper and SAQ-Web) if all online respondents are pooled and all paper-and-pencil respondents are pooled across all survey designs?

## Methods

### Study Design

The methodological pilot study used a sample of children and adolescents registered in the local resident registries of 20 municipalities in 5 federal states of Germany, covering urban and rural areas as well as the eastern and western regions of the country.

Data were collected using SAQ-Web or SAQ-paper methods. All selected individuals were invited by mail to participate in the study. They were sent a cover letter with the invitation to participate, information about the study and data privacy, and an informed consent form. Depending on the allocated mode, the invitation comprised a username and password for participation through the Web option along with a paper questionnaire for those allocated to the concurrent mixed-mode design, only a paper questionnaire in the single-mode design, or only the access data for the online questionnaire in the sequential mixed-mode design. The SAQ-Web questionnaire was only optimized for desktop computers. A reminder was sent by mail to respondents who had not replied within 3 weeks of the initial invitation. Participants who did not respond to the reminder were telephoned up to 5 times 4 weeks after the initial invitation. As an additional motivation for prospective participants, each parent and adolescent who had completed a questionnaire received a shopping voucher to the value of €10. The methodological pilot study strictly adhered to the data protection regulations set out in the German Federal Data Protection Act. Participation in the study was voluntary. All parents and participating adolescents were informed about the study’s aims and content, as well as data protection, and they provided informed consent. Following the strict data privacy protocol, prospective participants between the ages of 11 and 17 years received their questionnaires only after their parents provided consent.

Different questionnaires were used for different age groups. Main health indicators were included on the health questionnaires for parents of all age groups (0-17 years), and self-report data for main health indicators were obtained from adolescents aged 11-17 years. To reduce the risk of mode effects, the 2 questionnaires were designed to be as similar as possible and contained the same wording for the questions and response categories. On the basis of the unified-mode design [27], the wording and formatting of questions and response categories were standardized. To help participants visually distinguish single-choice questions from multiple-choice questions, all survey modes used the same checkbox design. Single-choice checkboxes were round, whereas multiple-choice checkboxes were rectangular. Additionally, multiple-choice questions included the instruction “Multiple entries are possible.” For filter questions, Web-based questionnaires were optimized with filter skips whenever the perceivability of the questions was not impaired. Plausibility checks and ranges were defined for the Web-based questionnaire. Additionally, soft prompting was programmed into the Web-based questionnaire to reduce item nonresponse. These differences were used to capitalize on the advantages of the Web mode for better data quality, and they were the only mode-specific design differences. Detailed information of the survey design and other technical aspects of the Web-based part of the survey are described in a “Checklist for Reporting Results of Internet E-Surveys” [28] (Multimedia Appendix 1).

**Figure 1:**
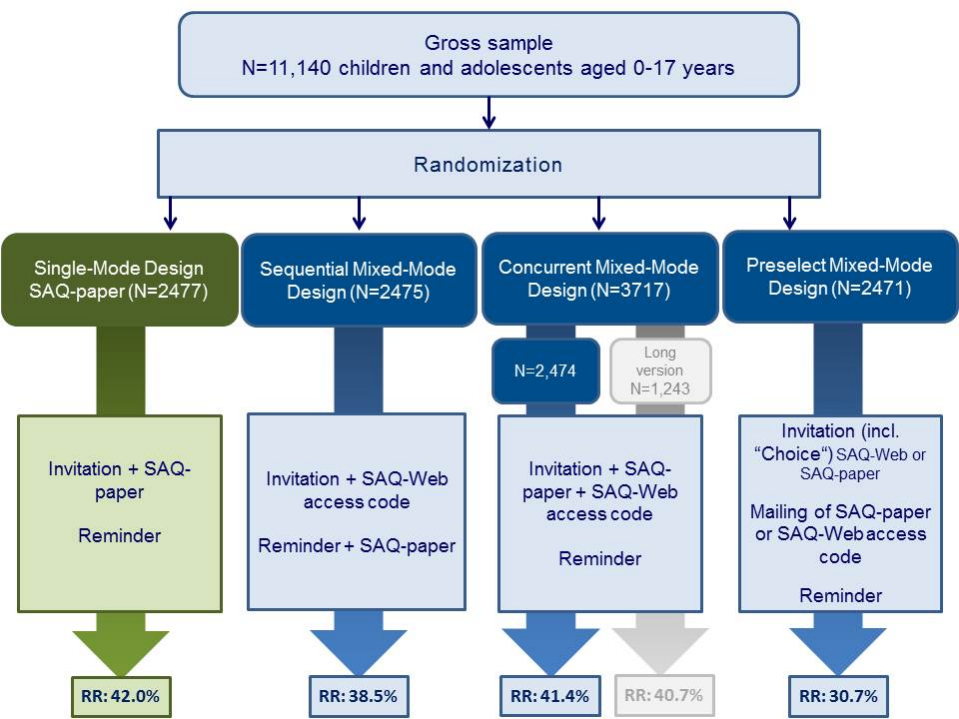
Study design of the methodological pilot study. SAQ-paper: self-administered paper-based questionnaire; SAQ-Web: self-administered Web-based questionnaire.

As shown in Figure 1, a gross sample of 11,140 children and adolescents was randomly allocated to four survey designs:

1. A single-mode survey design as a control group—respondents were sent an invitation letter and paper-and-pencil questionnaires, followed by a reminder after 3 weeks
2. A sequential mixed-mode survey design—respondents were sent an invitation letter and an online access code, followed 3 weeks later with a reminder letter and a paper-based questionnaire
3. A concurrent mixed-mode survey design—respondents were sent an invitation letter, a paper-based questionnaire, and an online access code (a longer version of the questionnaire was tested with a subgroup of the concurrent mixed-mode design, but this subgroup was excluded from this study) and
4. A preselect mixed-mode design—respondents were sent the invitation along with a postcard asking participants to choose one of the 2 options (SAQ-Web or SAQ-paper), followed by a reminder with the same offer

There were no statically significant differences in the (gross) sample composition across the 4 design groups in terms of known sample characteristics, such as age, sex, municipality size, region, or respondent citizenship, which were obtained from local registries.

The combined response rate for all survey designs was 38.43% (n=4032), following the internationally used Standard Definitions of Outcome Rates for Surveys of the American Association for Public Opinion Research (AAPOR Response Rate 2) [29]. There were no significant differences in response rates among the concurrent mixed-mode design, the sequential mixed-mode design, and the single-mode control group design. However, there was a significantly lower response rate in the preselect mixed-mode design. Detailed comparisons of response rates, sample compositions, data quality, and efforts among the different survey designs have been published previously [26].

### Database

For this study, only survey design groups using the same version of the questionnaire were included, with 3468 completed parent-reported health questionnaires for children and adolescents aged 0-17 years and 1194 questionnaires completed by adolescents aged 11-17 years. A response was defined as one completed health questionnaire from either parents or children. Hence, a valid response did not require both parents and children to complete all requested questionnaires. To answer the first research question regarding mode equivalence across the different survey designs, we compared the single-mode control group with each of the 3 mixed-mode groups. To answer the second research question regarding mode equivalence between the 2 data collection modes, data from all survey designs were pooled (Table 1).

### Sociodemographic Characteristics of Responding Parents and Adolescents by Survey Design and Data Collection Mode

#### Analyzed Sociodemographic Characteristics

The sample compositions of participating parents and adolescents were described by various sociodemographic characteristics separately by survey design and data collection mode. The variables examined included individual adolescent characteristics (age, sex, migration background, and highest level of education reached or aspired); parental characteristics (age, marital status, and participating parent); location (municipality size and region [East vs West Germany]); and household properties (education level and net household income). Household education level was measured using the Comparative Analysis of Social Mobility in Industrial Nations [30]. Household income was assessed using a question on household monthly net income.

**Table 1:** Cases used in this study.

Questionnaire type	Survey design										Mode group		
	Single-mode design	Sequential mixed-mode design			Concurrent mixed-mode design			Preselect mixed-mode design					
	SAQ-paper^a^	SAQ-paper	SAQ-Web^b^	Total	SAQ-paper	SAQ-Web	Total	SAQ-paper	SAQ-Web	Total	SAQ-paper	SAQ-Web	Total
Parent-completed health questionnaires	970	488	366	854	837	109	946	465	233	698	2760	708	3468
Adolescent-completed health questionnaires	343	117	152	269	244	46	290	191	101	292	895	299	1194

^a^SAQ-paper: self-administered paper-based questionnaire.

^b^SAQ-Web: self-administered Web-based questionnaire.

#### Statistical Methods

Differences between the control group and the different mixed-mode groups and between the 2 data collection modes were tested using chi-squared tests.

### Mode Equivalence of Health Indicators Between Survey Designs and Data Collection Modes

A wide range of health status indicators and health behaviors for children and adolescents with high public health relevance were analyzed to identify differences between the mixed-mode designs and the single-mode control group, as well as mode differences between SAQ-paper and SAQ-Web.

#### Analyzed Indicators of Physical and Mental Health

Lifetime diagnoses of asthma, hay fever, atopic eczema, and attention-deficit hyperactivity disorder (ADHD) were indicated by parents. Recurrent pain during the last 3 months was measured using the adolescents’ self-reports. Self-rated health (SRH) and chronic diseases were evaluated by parental report using the Minimum European Health Module questions [31], modified for children. Adolescents also answered the SRH question. Impairments owing to health problems were evaluated with a question from the Children with Special Health Care Needs Screener, which was answered by parents [32]. To define obesity, body mass index was calculated based on self-reported weight and height for adolescents and parent-reported weight and height for children aged 3-10 years. The body mass index cut-offs used in this study were determined by German norms [33].

Child and adolescent mental health problems were evaluated using the parent- and self-report Strengths and Difficulties Questionnaire (SDQ) [34]. An SDQ total difficulties score was calculated for all children and adolescents. Participants with a borderline or abnormal score (based on German norms) [35] were defined as at risk for emotional and behavioral symptoms. Participants with borderline or abnormal SDQ impact scores were defined as at risk for psychosocial impairment.

#### Analyzed Indicators of Health Care Utilization

As indicators of health care use, pediatrician and orthodontist visits during the past 12 months for adolescents and parent-reported visits to any doctor for children under 11 years were analyzed [36].

#### Analyzed Measure of Health-Related Quality of Life

Health-related quality of life (HRQoL) was measured using KIDSCREEN-27 for adolescents aged 11-17 years, with 5 subscores for physical and psychological well-being, relationships with peers and parents, and school well-being. Scores were summed and transformed into *t* values [37].

#### Analyzed Health Behaviors

Adolescents reported their current smoking status, water pipe consumption during the past 12 months, second-hand smoke exposure [38], and lifetime consumption and current use of screen-based media. Excessive use of screen-based media was defined as more than 2 hours per day [39]. Harmful alcohol use and binge drinking were defined using responses to the Alcohol Use Disorders Identification Test (AUDIT-C) [40].

Following the recommendation of the World Health Organization [41], healthy physical activity was defined as physical activity for at least 60 min per day. Low physical activity was defined as less than 2 days per week of at least 60 min of activity. All questions on physical activity were answered by adolescents aged 11-17 years.

#### Statistical Methods

We calculated prevalence rates for dichotomous health indicators and mean values for HRQoL (a scale outcome) by survey design and data collection mode. We compared these values using *z* or *t* tests.

Due to the different sample compositions of the SAQ-paper and SAQ-Web groups (see the Results), it was necessary to control for sociodemographic characteristics to identify possible mode effects. Survey modes can differ in selection (different population groups prefer different modes) and measurement (different answers are given by the same person under different modes of administration), so these differences are confounded [42]. Additionally, health status and health behavior differ by sex, education, and other sociodemographic characteristics [43,44]. To eliminate the risk of confounding, we adjusted for sociodemographic characteristics by calculating adjusted prevalence rates using predictive margins [45] based on logistic or linear regression models with sociodemographic factors as covariates. To analyze indicators based on parental reports, we included child attributes (age, sex, and migration background); parental attributes (relationship to the child, age, and marital status); household attributes (education and income); and regional attributes (region and municipality size). Adolescents’ reports were adjusted by child attributes, including the highest level of education completed, as well as household attributes and location. The mode of data collection was another covariate used to identify adjusted prevalence for each mode. Differences were tested using *z* or *t* tests.

For the survey design comparison, crude as well as adjusted prevalence rates and mean values were calculated. A statistical test for diversity was conducted between the single-mode control group design and each of the 3 mixed-mode designs. Because the survey design samples did not differ in sociodemographic characteristics (see the Results) and there were only marginal differences between the 2 approaches, only the results for crude prevalence rates or mean values without adjustment for sociodemographic characteristics to simplify the presentation of results are shown here.

#### Handling of Multiple Testing

In total, we analyzed 12 health indicators using the parental sample and 28 using the adolescent sample. For these health indicators, we tested each mixed-mode survey design against the control group. Additionally, we used 2 other statistical tests to identify differences between the data collection modes, using first the crude values and then the adjusted values.

Regarding the research questions, a sensitive approach to detect possible differences (ie, a higher probability of accepting the null hypothesis) is needed. Therefore, we decided to address the statistical problem of multiple testing by correcting the significance level only for the number of tests performed for each health indicator. This was done only for tests comparing the different survey designs. We used the Bonferroni correction method to neutralize the accumulation of α-error [46], using an adjusted significance level of *P*<.02 to examine differences between the mixed-mode survey designs and the single-mode control group. For the comparison of data collection modes, a significance level of α=.05 was used.

## Results

### Sociodemographic Characteristics of Responding Parents and Adolescents in Different Survey Designs and Different Data Collection Modes

#### Responding Parents

There were no statistically significant differences in sample composition between the mixed-mode survey designs and the single-mode control group for participating parents. However, the sample sociodemographic characteristics differed significantly between data collection modes (Multimedia Appendix 2). Parents responding online were more often married and had higher household education levels, higher incomes, and younger children than those who responded to the SAQ-paper. More fathers responded via the Web-based questionnaire than in the paper-and-pencil group. There were no significant differences in migration background, parental age. *P* values close to significant level are found for region of residence (*P*=.08), municipally size (*P*=.05) or child’s sex (*P*=.06).

#### Responding Children and Adolescents

For the responding children and adolescents (aged 11-17 years), there were no statistically significant differences in sociodemographic characteristics between the different survey designs, but adolescents responding online were more often male, had reached or aspired to reach higher levels of education, and were more likely to live in households with higher education and higher income, compared with adolescents who responded to the SAQ-paper (Multimedia Appendix 3).

### Mode Equivalence of Health Indicators Between Survey Designs and Data Collection Modes

#### Physical and Mental Health

The analyzed indicators of physical and mental health status showed no statistically significant differences by survey design or data collection mode (Table 2). Across modes and designs, parents reported the same results for SRH, chronic disease, impairment owing to health problems, lifetime prevalence of diagnosed diseases, obesity, and mental health problems and impairment. Adolescent self-reports showed no statistically significant differences in SRH, mental health problems and impairment, or chronic pain.

#### Health Care Utilization

No differences in the crude or adjusted prevalence rates were found in adolescent-reported 12-month use of pediatric or orthodontic services (Table 3). The crude prevalence of parent-reported 12-month use of any doctor and of pediatric services (for children under 11 years) differed significantly, with more frequent reports of doctor’s visits in the SAQ-Web group. After adjusting for sociodemographic attributes, this difference disappeared. There were no significant differences between the mixed-mode design groups and the control group for any of the analyzed indicators of health care utilization.

#### Health-Related Quality of Life

HRQoL, measured using the 5 dimensions of the KIDSCREEN-27 for adolescents, was the only indicator scale analyzed. Independent of adjustment, there were no significant differences between the 2 data collection modes (SAQ-paper and SAQ-Web) for any of the observed dimensions (Table 4). Regarding survey design, better psychological well-being was reported in the concurrent mixed-mode design and better relations with parents were reported in the preselect mixed-mode survey design, compared with the single-mode control group. After correcting the significance level for multiple testing, no differences were found by survey design.

#### Health Behaviors

The crude prevalence of lifetime alcohol consumption (self-reported by adolescents aged 11-17 years), as well as hazardous consumption and binge drinking (based on AUDIT-C reports), showed significant differences between SAQ-paper and SAQ-Web, with higher levels of alcohol consumption reported by online participants (Table 5). Although the differences in hazardous consumption and binge drinking between the 2 modes of data collection disappeared after controlling for sociodemographic characteristics, significantly more online respondents than paper-and-pencil respondents reported that they had consumed alcohol.

There were no differences in other health behaviors assessed (tobacco consumption, physical activity, and media consumption) by survey design or data collection mode.

**Table 2:** Physical and mental health status of children and adolescents aged 0-17 years by survey design and data collection mode (prevalence rates).

Physical and mental health status		Survey design^a^										Data collection mode^a^									
		Single-mode design	Sequential MM^b^ design		Concurrent MM^b^ design			Preselect MM^b^ design			SAQ-Web^c^ (crude)			SAQ-paper^d^ (crude)			SAQ-Web^c^ (adjusted^e^)		SAQ-paper^d^ (adjusted^e^)		
		n (%)	n (%)	*P*^f^		n (%)	*P*^f^		n (%)	*P*^f^			n (%)		n (%)	*P*		n (%)		n (%)	*P*
**General health status**																					
	Self-rated health (very good, good)^g^	964 (97.6)	848 (97.1)	.46		942 (97.3)	.71		693 (97.3)	.65			708 (97.0)		2739 (97.41)	.60		708 (96.7)		2739 (97.48)	.31
	Self-rated health (very good, good)^h^	338 (90.2)	267 (92.1)	.41		286 (87.1)	.22		290 (92.8)	.26			299 (90.6)		881 (90.5)	.93		299 (90.1)		881 (90.6)	.81
	Chronic disease (yes)^g^	962 (10.2)	849 (10.7)	.71		941 (7.4)	.03^e^		691 (9.6)	.67			708 (10.2)		2735 (9.25)	.47		708 (10.5)		2735 (9.18)	.31
	Impairment owing to health problems (yes)^g^	956 (3.2)	851 (4.0)	.39		937 (3.4)	.83		692 (3.6)	.69			708 (4.1)		2728 (3.41)	.40		708 (4.5)		2728 (3.36)	.19
**Allergies**																					
	Bronchial asthma (lifetime diagnosis)^g^	951 (4.5)	838 (6.1)	.14		928 (4.6)	.91		675 (5.2)	.54			701 (4.6)		2691 (5.20)	.48		701 (5.0)		2691 (5.10)	.91
	Neurodermatitis (lifetime diagnosis)^g^	951 (15.0)	839 (16.0)	.59		927 (13.8)	.45		678 (17.4)	.20			704 (17.0)		2691 (14.98)	.19		704 (17.5)		2691 (14.95)	.12
	Hay fever (lifetime diagnosis)^g^	955 (11.2)	831 (11.2)	.99		931 (10.7)	.75		680 (11.5)	.87			702 (11.3)		2695 (11.09)	.91		702 (12.0)		2695 (10.96)	.50
**Chronic pain**																					
	Headache (recurrent during the last 3 months)^h^	331 (35.6)	260 (37.7)	.61		274 (33.9)	.66		282 (31.2)	.24			299 (32.1)		847 (35.4)	.30		299 (33.8)		847 (34.8)	.77
	Dorsal pain (recurrent during the last 3 months)^h^	321 (25.9)	257 (23.0)	.42		265 (24.2)	.64		276 (27.5)	.64			298 (22.5)		820 (26.2)	.19		298 (23.6)		820 (25.8)	.45
	Any pain (recurrent during the last 3 months)^h^	343 (84.3)	265 (83.0)	.68		288 (81.9)	.44		291 (81.1)	.30			294 (82.3)		892 (82.7)	.87		294 (82.6)		892 (82.6)	.99
**Mental health problems**																					
	Attention-deficit hyperactivity disorder (lifetime diagnosis)^g^	824 (5.1)	716 (4.1)	.33		794 (5.0)	.96		593 (3.2)	.07			595 (3.5)		2332 (4.67)	.19		595 (3.9)		2332 (4.56)	.45
	At risk for emotional and behavioral symptoms^g^	788 (13.6)	695 (15.5)	.29		754 (13.7)	.96		577 (12.7)	.62			598 (13.0)		2216 (14.12)	.49		598 (13.9)		2216 (13.89)	.97
	At risk for emotional and behavioral symptoms^h^	331 (9.7)	255 (12.2)	.34		278 (14.0)	.10		283 (14.1)	.09			293 (13.7)		853 (12.0)	.46		293 (13.)		853 (11.9)	.41
	At risk for impairment following psychosocial problems^g^	820 (18.5)	719 (17.4)	.56		789 (17.2)	.50		592 (16.6)	.33			600 (16.3)		2320 (17.80)	.39		600 (17.5)		2320 (17.48)	.99
	At risk for impairment following psychosocial problems^h^	339 (16.5)	263 (19.0)	.43		286 (16.4)	.98		288 (18.1)	.61			298 (19.8)		877 (16.5)	.21		298 (19.5)		877 (16.6)	.30
**Obesity**																					
	Obesity of children (aged 0-10 years)^g^	401 (2.7)	407 (2.7)	.97		423 (2.4)	.73		302 (4.0)	.38			370 (1.9)		1163 (3.18)	.14		370 (2.7)		1163 (3.49)	.46
	Obesity of adolescents (aged 11-17 years)^h^	330 (5.2)	260 (4.6)	.76		275 (4.4)	.65		275 (3.6)	.36			291 (3.4)		848 (4.8)	.28		291 (4.1)		848 (4.6)	.78

^a^Sample sizes are shown in Table 1.

^b^MM: mixed-mode.

^c^SAQ-Web: self-administered Web-based questionnaire.

^d^SAQ-paper: self-administered paper-based questionnaire.

^e^Adjusted for age of the child (adolescent, parent); sex of the child (adolescent); relationship to the child (parent); household income (adolescent, parent); parental education (adolescent, parent); adolescent education (adolescent); region (adolescent, parent); municipality size (adolescent, parent); and parental marital status (parent).

^f^Tested against single-mode control group.

^g^Proxy-reported by parents of children and adolescents aged 0-17 years.

^h^Self-reported by adolescents aged 11-17 years.

**Table 3:** Health care utilization among children and adolescents aged 0-17 years by survey design and data collection mode (prevalence rates).

Health care utilization		Survey design^a^							Data collection mode^a^					
		Single-mode design	Sequential MM^b^design		Concurrent MM^b^design		Preselect MM^b^design		SAQ-Web^c^ (crude)	SAQ-paper^d^ (crude)		SAQ-Web^c^ (adjusted^e^)	SAQ-paper^d^ (adjusted^e^)	
		n (%)	n (%)	*P*^f^	n (%)	*P*^f^	n (%)	*P*^f^	n (%)	n (%)	*P*	n (%)	n (%)	*P*
**Medical care use**														
	Any doctor (children aged 0-13 years; past 12 months)^g^	970 (91.3)	804 (92.7)	.31	935 (91.8)	.74	645 (91.5)	.93	594 (95.3)	2760 (91.05)	<.001	594 (92.2)	2760 (91.69)	.75
	Pediatric services (children aged 0-13 years; past 12 months)^g^	970 (70.0)	803 (71.6)	.46	935 (72.0)	.34	645 (71.6)	.48	593 (80.9)	2760 (69.16)	<.001	593 (70.9)	2760 (71.47)	.75
	Pediatric services (adolescents aged 14-17 years; past 12 months)^h^	205 (35.6)	147 (35.4)	.96	155 (29.0)	.18	159 (35.8)	.96	164 (34.1)	502 (34.1)	.99	164 (33.8)	502 (34.2)	.92
	Orthodontic services (adolescents aged 14-17 years; past 12 months)^h^	332 (40.4)	260 (37.3)	.45	281 (39.5)	.83	288 (44.4)	.31	295 (39.7)	865 (40.7)	.75	295 (40.3)	865 (40.5)	.96

^a^Sample sizes are shown in Table 1.

^b^MM: mixed-mode.

^c^SAQ-Web: self-administered Web-based questionnaire.

^d^SAQ-paper: self-administered paper-based questionnaire.

^e^Adjusted for age of the child (adolescent, parent); sex of the child (adolescent); relationship to the child (parent); household income (adolescent, parent); parental education (adolescent, parent); adolescent education (adolescent); region (adolescent, parent); municipality size (adolescent, parent); and parental marital status (parent).

^f^Tested against single-mode control group.

^g^Proxy-reported by parents of children and adolescents aged 0-17 years.

^h^Self-reported by adolescents aged 11-17 years.

**Table 4:** Health-related quality of life of adolescents aged 11-17 years by survey design and data collection mode (mean values).

Health related quality of life		Survey design^a^								Data collection mode^a^							
		Single-mode design	Sequential MM^b^design		Concurrent MM^b^design		Preselect MM^b^design		SAQ-Web^c^ (crude)		SAQ-paper^d^ (crude)			SAQ-Web^c^ (adjusted^e^)		SAQ-paper^d^ (adjusted^e^)	
		n (%)	n (%)	*P*^h^	n (%)	*P*^h^	n (%)	*P*^h^	n (%)		n (%)	*P*	n (%)		n (%)		*P*
**Dimensions**																	
	Physical well-being (mean)^f^	337 (49.9)	263 (49.9)	.98	282 (49.2)	.38	290 (49.0)	.21	297 (49.5)		874 (49.5)	.95	297 (49.0)		874 (49.7)		.26
	Psychological well-being (mean)^f^	336 (51.2)	265 (50.0)	.14	283 (49.6)	.05^g^	291 (49.8)	.07	297 (49.6)		877 (50.4)	.20	297 (49.3)		877 (50.5)		.07
	Relations with parents (mean)^f^	331 (53.5)	263 (53.0)	.52	283 (53.3)	.74	290 (52.0)	.04^g^	296 (52.5)		871 (53.1)	.31	296 (52.3)		871 (53.2)		.18
	Relations with peers (mean)^f^	340 (51.2)	266 (50.1)	.14	288 (50.5)	.37	292 (50.1)	.13	297 (49.8)		888 (50.8)	.12	297 (49.9)		888 (50.7)		.18
	Well-being in school (mean)^f^	335 (51.9)	265 (51.6)	.66	283 (50.8)	.08	285 (51.4)	.43	294 (51.1)		873 (51.60)	.31	294 (50.9)		873 (51.70)		.17

^a^Sample sizes are shown in Table 1.

^b^MM: mixed-mode.

^c^SAQ-paper: self-administered paper-based questionnaire.

^d^SAQ-Web: self-administered Web-based questionnaire.

^e^Adjusted for age of the child (adolescent, parent); sex of the child (adolescent); relationship to the child (parent); household income (adolescent, parent); parental education (adolescent, parent); adolescent education (adolescent); region (adolescent, parent); municipality size (adolescent, parent); and parental marital status (parent).

^f^Self-reported by adolescents aged 11-17 years.

^g^Not significant, *P* value adjusted with Bonferroni correction.

^h^Tested against single-mode control group.

**Table 5:** Health behaviors of adolescents aged 11-17 years by survey design and data collection mode (prevalence-rates).

Health related quality of life		Survey design^a^							Data collection mode^a^						
		Single-mode design	Sequential MM^b^ design		Concurrent MM^b^ design		Preselect MM^b^ design			SAQ-Web^c^ (crude)	SAQ-paper^d^ (crude)		SAQ-Web^c^ (adjusted^e^)	SAQ-paper^d^ (adjusted^e^)	
		n (%)	n (%)	*P*^i^	n (%)	*P*^i^	n (%)	*P*^i^		n (%)	n (%)	*P*	n (%)	n (%)	*P*
**Tobacco consumption**															
	Current smoking status (yes)^f^	342 (8.8)	264 (8.3)	.85	288 (11.5)	.27	291 (10.7)	.43		295 (11.5)	889 (9.2)	.27	295 (11.6)	889 (9.2)	.25
	Water pipe consumption (past 12 months, yes)^f^	339 (18.0)	262 (17.9)	.99	286 (18.2)	.95	291 (16.2)	.54		295 (20.3)	882 (16.7)	.17	295 (19.1)	882 (17.1)	.38
	Second-hand smoke exposure (yes)^f^	308 (13.6)	239 (15.5)	.55	249 (10.8)	.31	259 (14.3)	.82		261 (11.1)	793 (14.4)	.16	261 (12.9)	793 (13.8)	.74
**Alcohol consumption**															
	Lifetime consumption of alcohol (yes)^f^	343 (51.0)	262 (56.1)	.21	290 (50.7)	.93	292 (54.8)	.34		295 (61.4)	891 (50.3)	.001	295 (60.1)	891 (50.7)	<.001
	Hazardous alcohol consumption (based on AUDIT-C^g^)^f^	328 (12.2)	255 (12.5)	.90	284 (10.6)	.53	284 (12.7)	.86		293 (17.1)	857 (10.3)	.01	293 (14.9)	857 (11.9)	.16
	Binge drinking (based on AUDIT-C)^f^	339 (7.7)	262 (7.7)	.99	288 (5.6)	.29	291 (8.9)	.57		295 (10.5)	884 (6.5)	.04	295 (10.0)	884 (7.2)	.13
**Physical activity**															
	Physical activity consistent with WHO^h^ guidelines^f^	343 (6.1)	265 (5.3)	.66	288 (3.1)	.07	292 (3.8)	.17		295 (4.1)	892 (4.8)	.58	295 (3.3)	892 (5.2)	.16
	Low physical activity^f^	343 (14.9)	265 (12.5)	.39	288 (15.3)	.89	292 (17.1)	.44		295 (13.9)	892 (15.2)	.57	295 (14.5)	892 (15.0)	.82
	Currently doing sports^f^	342 (79.5)	265 (81.9)	.46	286 (78.0)	.63	291 (77.0)	.44		295 (80.3)	888 (78.6)	.52	295 (79.3)	888 (78.9)	.89
**Media consumption**															
	Social media (>2 hours/day)^f^	341 (20.2)	262 (19.1)	.72	289 (17.3)	.35	290 (19.7)	.86		295 (20.0)	886 (18.8)	.67	295 (21.3)	886 (18.4)	.30
	TV (>2 hours/day)^f^	341 (42.2)	262 (41.6)	.88	289 (41.2)	.79	292 (38.0)	.28		294 (39.1)	889 (41.4)	.49	294 (41.5)	889 (40.6)	.79
	Game console (>2 hours/day)^f^	339 (18.9)	261 (19.9)	.75	287 (18.5)	.90	290 (12.8)	.03^i^		295 (20.0)	881 (16.7)	.21	295 (19.9)	881 (16.7)	.22

^a^Sample sizes are shown in Table 1.

^b^MM: mixed-mode.

^c^SAQ-Web: self-administered Web-based questionnaire.

^d^SAQ-paper: self-administered paper-based questionnaire.

^e^Adjusted for age of the child (adolescent, parent); sex of the child (adolescent); relationship to the child (parent); household income (adolescent, parent); parental education (adolescent, parent); adolescent education (adolescent); region (adolescent, parent); municipality size (adolescent, parent); and parental marital status (parent).

^f^Self-reported by adolescents aged 11-17 years.

^g^AUDIT-C: Alcohol Use Disorders Identification Test.

^h^WHO: World Health Organization; sample sizes are shown in Table 1.

^i^Not significant, *P* value adjusted with Bonferroni correction.

## Discussion

### Summary

The main aim of this study was to examine the risk of mode effects in a mixed-mode health interview survey for children and adolescents that combined paper-and-pencil questionnaires and Web-based questionnaires. Therefore, we compared prevalence rates and mean values of a broad range of health indicators from 3 alternative mixed-mode designs (all combining paper-and-pencil and Web-based questionnaires) with a single-mode control group (paper-and-pencil only). We also compared results between online respondents and paper-and-pencil respondents regardless of the survey design. First, we examined differences in sociodemographic characteristics by survey design and data collection mode, as it is well documented that sociodemographic characteristics are associated with health status and health behavior [43,44]. Regarding survey design, there were no statistically significant differences in sample composition, prevalence rates, or mean values of the examined health indicators. There were differences in sociodemographic characteristics across the data collection mode groups. After adjusting for these differences, only one of the analyzed health indicators (lifetime alcohol consumption) showed between-group differences. These results indicate that there is limited mode bias in health interview surveys for children and adolescents using a mixed-mode design with Web-based and paper questionnaires.

### Sample Composition and Digital Divide

Consistent with previous findings, the sample composition of responding parents and of responding adolescents differed by data collection mode. We confirmed the so-called “digital divide” [47-50]—male adolescents and younger fathers preferred the online mode, a well-known systematic difference [5] between these modes[10,49,51-54]. Additionally, SAQ-Web respondents had higher household incomes [15,49,55] and higher household education levels [10,49,54-57]. Despite these differences, and differences in online response rates between the mixed-mode survey designs, there were no statistically significant differences in sample composition between the paper-and-pencil single-mode control group and the 3 mixed-mode groups. To control for the influence of sociodemographic on health indicators, we adjusted for sociodemographic characteristics by first calculating crude prevalence rates. Then, the analysis was complemented with adjusted prevalence rates or adjusted mean values using predictive margins to identify possible mode effects. Comparisons between the mixed-mode survey designs and the single-mode control group were made using only the crude prevalence rates. Using this approach, hardly any statistically significant differences by data collection mode or by survey design were found for the analyzed health indicators.

### Health Status and Health Care Utilization

Prevalence rates of health complaints, such as diagnosed allergies, diagnosed ADHD, obesity, and chronic pain, were equivalent between the modes, as previous studies of adults [9,11,53,58] and adolescents [12] have shown. A population-based Norwegian study found higher asthma prevalence rates among online respondents; this was interpreted as possible nonresponse bias and not as a mode effect because there were no differences in the prevalence rates for any other condition [59]. A literature review by Hox et al showed that after controlling for selection, small mode effects do appear, most often distinguishing between modes that involve interviewers (face-to-face, telephone) and modes that do not (mail, Web) [42].

We found similar prevalence rates for SRH, chronic diseases, and impairment owing to health problems between SAQ-paper and SAQ-Web respondents. The 2 previous studies examining these health indicators among adults in general [11] and among older adults [10] also found no differences between these 2 data collection modes. Another study of adults interpreted the higher SRH found among online respondents compared with paper-based respondents as an expression of different sample characteristics linked to the digital divide era [49], or a case of better-situated people with better health using Web-based questionnaires, and not as a mode effect. We cannot say whether this holds true for the KiGGS methodological pilot study, because we controlled for most characteristics linked to the preference for online participation, such as region of residence and education or income.

For mental and psychosocial problems, we calculated risk groups for emotional and behavioral problems and for impairment owing to psychosocial problems based on SDQ scores [34]. Both parent- and adolescent-reported scores were equivalent across the examined modes. Several other studies have postulated the comparability of measurement results between these 2 self-administered modes for other standardized mental health questionnaires (eg, depression or anxiety) [12,58,60,61].

In their review of 55 studies investigating 79 instruments, Campbell et al [9] found measurement equivalence for electronic- and paper-based patient outcomes and concluded that standardized instruments can generally be used electronically without measurement effects. In our study, we also found comparable results for standardized instruments (the SDQ and AUDIT-C), as well as for self-reported HRQoL (KIDSCREEN-27). No existing studies have compared these particular instruments, but previous studies have compared the Short Form Health Survey-36, a frequently used standardized HRQoL instrument for adults, and found measurement equivalence [9,58,62-64].

All reports of health care utilization were equivalent between the self-administered modes; this is consistent with prior empirical results, including studies of adult vaccination use [11], adolescent health care use [12], and multiple health care quality indicators [56]. The greater use of pediatric services (and of any doctor) before adjustment for sociodemographic characteristics may be explained by the younger age of children in the online group—in Germany, all children are invited to undergo regular health screening examinations (U3-U9 examinations) from early childhood until the age of 5 years, with a well-established system of reminders and reporting.

### Health Behaviors

Most of the analyzed adolescent health behaviors (current smoking, 12-month water pipe consumption, second-hand smoke exposure, physical activity, and screen-based media use) showed comparable results and no differences between the 2 modes. These results are consistent with the results of other studies on adolescents [12,65].

Considering alcohol consumption, the crude and adjusted prevalence rates for lifetime consumption were significantly higher among SAQ-Web-responding adolescents. After adjusting for sociodemographic characteristics, the difference decreased but could not be explained by the sociodemographic differences between the 2 groups of respondents. The prevalence of hazardous consumption and binge drinking were comparable between data collection modes after controlling for sample composition.

Most previous studies have reported no statistically significant differences in alcohol consumption among adolescents or young adults by these 2 data collection modes [12,66]. However, research comparing sensitive health behaviors is inconsistent. Some studies have found higher adult binge drinking [53] and higher adolescent alcohol consumption [17] in online reports, whereas others have found no difference in sensitive health behaviors in general for college students [67,68] and young adults [69].

The higher rate of reported lifetime alcohol consumption among SAQ-Web-responding adolescents, in the absence of frequently reported hazardous consumption or binge drinking, may be interpreted in multiple ways. For example, this may be a result of different sample properties, such as SAQ-Web-preferring adolescents being more likely to experiment with alcohol consumption. However, it is also possible that this result is a mode effect based on the assumption of identical alcohol consumption in both groups. Web-based questionnaires afford greater privacy because there is no risk of parents checking the responses. Another possible explanation is the lower social orientation in the Internet mode [13]. Both these explanations assume that Web-based questionnaires are more likely to elicit honest reports, but the similar results between the 2 mode groups for reported harmful alcohol consumption after adjustment contradict this assumption. Taken together, the results for alcohol consumption suggest that lifetime consumption should be used with caution as a health indicator in a mixed-mode design. Hazardous consumption and binge drinking are better indicators because they exhibit mode equivalence and have greater public health relevance than lifetime consumption, which is measured by a single question asking whether the respondent has ever consumed alcohol.

### Main Result

Other empirical comparisons of measurement results between different mixed-mode survey designs are rare. In accord with one other result for the adult population [70], all of the analyzed health indicators for children and adolescents showed comparable results, with no statistically significant differences between the single-mode control group and the 3 mixed-mode groups. Additionally, sociodemographic characteristics did not differ by survey design for parents or adolescents. Regarding measurement comparability, any of the tested mixed-mode health interview survey designs, which offer both Web-based and paper questionnaires, could be used for children and adolescents.

### Strengths and Limitations

The strengths of the methodological pilot study are the randomized study design, the population-based sample, and the inclusion of a single-mode control group as a reference to interpret the results. However, there are also some limitations, predominantly the relatively small size of the net samples of the analyzed groups. Each survey design had a relatively low number of cases, so interpretations of the results based on the net samples must be made with caution. Possible differences across the 4 survey designs or between the 2 data collection modes could have been overlooked because of a lack of statistical power, particularly regarding the need for correction for multiple testing. Other limitations concern the external validity of the results; the study was conducted in a German setting using register-based samples of children and adolescents, so the results are difficult to generalize to other countries, settings, or populations.

### Conclusions

Our results are consistent with those of most previous studies. We found comparable results between the 2 self-administered modes (SAQ-Web and SAQ-paper) for almost all analyzed health indicators, except for lifetime consumption of alcohol among adolescents aged 11-17 years. Thus, no differences were found between the single-mode control group design and 3 mixed-mode survey designs that combined the 2 data collection modes.

These results suggest that it is possible to measure health indicators for children and adolescents using a mixed-mode design combining SAQ-Web and SAQ-paper methods, with a low risk of mode effects and high comparability across different mixed-mode survey designs combining these 2 data collection modes [4]. The implementation of a Web-based option in the existing paper-based interview surveys of children and adolescents has a low risk of changed measurement values caused by the mixed-mode survey design.

## Appendix

Multimedia Appendix 1 Checklist for Reporting Results of Internet E-Surveys (CHERRIES).

Multimedia Appendix 2 Sociodemographic characteristics of responding parents of children aged 0-17 years by survey design and data collection mode mode.

Multimedia Appendix 3 Sociodemographic characteristics of responding adolescents aged 11-17 years by survey design and data collection mode.

## Abbreviations

ADHD: attention-deficit hyperactivity disorder
AUDIT-C: Alcohol Use Disorders Identification Test
HRQoL: health-related quality of life
KiGGS: The German Health Interview and Examination Survey for Children and Adolescents
MM: mixed-mode
SAQ-paper: self-administered paper-based questionnaires
SAQ-Web: self-administered Web-based questionnaires
SDQ: Strengths and Difficulties Questionnaire
SRH: self-rated health
