# Supplementary material for: Mode Equivalence of Health Indicators Between Data Collection Modes and Mixed-Mode Survey Designs in Population-Based Health Interview Surveys for Children and Adolescents: Methodological Study
Source: J Med Internet Res. 2018 Mar 5;20(3):e64. doi: 10.2196/jmir.7802 (PMC5859740; doi:10.2196/jmir.7802)
Supplement: Multimedia Appendix 1 [file jmir_v20i3e64_app1.pdf]

Checklist for Reporting Results of Internet E-Surveys (CHERRIES) [1]

Item Category	Checklist Item	Methodological pilot study
**Design**		
Describe survey design		The methodological pilot study used a sample of children and adolescents registered in the local resident registries of twenty municipalities in five federal states of Germany, covering urban and rural areas as well as the eastern and western regions of the country.<br>As shown in Figure 1, a gross sample of 11,140 was randomly allocated to four survey designs:<br><br>(1) a single-mode survey design as a control group, where only paper-and-pencil questionnaires were sent to the respondents together with the invitation letter, followed by a reminder after three weeks,<br><br>(2) a sequential mixed-mode survey design, where an online access code was sent along with the invitation letter, followed three weeks later with a reminder letter and a paper-based questionnaire;<br><br>(3) a concurrent mixed-mode survey design, where a paper-based questionnaire and an online access code were sent to participants together with an invitation letter. (A long version of the questionnaire was tested among a subgroup of the concurrent mixed-mode design, but this subgroup is excluded from the present study);<br><br>(4) a pre-select mixed-mode design, where only the invitation was sent to the participants together with a postcard asking them to choose one of two options (SAQ-Web or SAQ-Paper), followed by a reminder with the same offer.
**IRB (Institutional Review Board) approval and informed consent process**		
IRB approval		For the methodological pilot study, we received a vote in favor by the Federal Commissioner for Data Protection and Freedom of Information. (Reference: III-401/008#0010). A vote by the IRB was for a health interview survey was upon consultation not necessary.
Informed consent and data protection		The methodological study was conducted by the German Public Health Institute (Robert-Koch Institute, RKI) from August to December 2013 as a part of the pretest of the third wave of the “German Health Interview and

Item Category	Checklist Item	Methodological pilot study
		Examination Survey for Children and Adolescents” (KiGGS).<br>The methodological pilot study as a part of the pretest of KiGGS wave 2aimed to compare three mixed-mode survey designs using web and paper-based questionnaires with a single-mode SAQ-Paper design in terms of response rates, sample composition, data quality, and costs. The pilot study also aimed to explore whether estimates of health indicators differed among the survey designs and data collection modes.<br>As all of the RKI’s surveys the methodological pilotstudy strictly observe the data protection regulations set out in the German Federal Data Protection Act. Participation in the study was voluntary. All parents and participating adolescents were informed about the study’s aims and content, as well as data protection, and provided their informed consent.
**Development and pre-testing**		
Development and testing		The survey was developed over several months. Before invitations were sent out to the prospective participants, the functionality of filters, ranges and transfer to databases was tested regularly.
**Recruitment process and description of the sample having access to the questionnaire**		
Open survey versus closed survey		Closed survey
Contact mode		The initial contact (invitation letter with login code) was sent by mail.
Advertising the survey		Closed survey without advertising
**Survey administration**		
Web/E-mail		Self-administered web questionnaires (desktop versions) with a mixed-mode survey offering paper-and-pencil questionnaires and/or online questionnaires
Context		For the KiGGS pilot study, a random sample was requested drawn from different German municipalities and cities. Prospective participants received via post a link including a log-in-code to a governmental project-owned website. As a restriction, only randomly selected prospective participants could enter the log in code.
Mandatory/voluntary		Voluntary survey

Item Category	Checklist Item	Methodological pilot study
Incentives		10 Euro shopping voucher/ questionnaire
Time/Date		August 2013 to December 2013
Randomization of items or questionnaires		No, fixed. Order like the paper-and-pencil questionnaires
Adaptive questioning		To reduce the risk of mode effects, the two questionnaires were designed to be as identical as possible, and the wording of the questions and answer categories did not differ. According to the Unified-Mode-Designs [22] the wording and formatting of questions and answering categories were standardized. To be able to distinguish visually single choice questions from multiple choice questions, checkboxes were designed identically for all survey modes. Single choice checkboxes were desgined in a round shape, whereas multiple choice checkboxes exhibit a rectangular shape. Moreover, in multiple choice questions participants were informed that: "Multiple entries are possible". For filter questions, online questionnaires were optimized with filter skips whenever the perceivability of the questions was not impaired. Plausibility checks and ranges were defined for the online questionnaire. Additionally, soft prompting was programmed in the online questionnaires to reduce item nonresponse. These differences were used to capitalize on the advantage of the web mode for better data quality and were the only mode-specific design differences.
Number of Items		What was the number of questionnaire items per page? The number of items is an important factor for the completion rate.<br>First of all, the online questionnaire and the paper and pencil questionnaire were designed in the same manner. The number of items per page differed and depended strongly on the indicators used. To minimize the use of scrolling in the online questionnaire, the number of items was derived from the paper and pencil questionnaire. Furthermore, different questionnaires for the different age groups were implemented.
Number of screens (pages)		Over how many pages was the questionnaire distributed? The number of items is an important factor for the completion rate.<br>Different questionnaires for the different age groups were implemented. Those questionnaires had a varying length and were dependent of the number of filter skips.
Completeness check		The informed consent, birth month, birth year and sex were forced choices and therefore checked for

Item Category	Checklist Item	Methodological pilot study
		completeness. This was used for data protection reasons and identification of the correct participant. All other items were voluntary, but soft prompting was used.<br>In some questions, the answer options “don’t know” or “rather not say” was offered as an answering category.
	Review step	Respondents had the possibility to use a Back button and change/ correct their answers. A summary responses has not been implemented.<br>Once, the questionnaire was completed, participants could not access their questionnaire again.
**Response rates**		
Unique site visitor		Due to data protection regulations, information on IP addresses and/ or cookies could not be stored.
View rate (Ratio of unique survey visitors/unique site visitors)		see above
Participation rate (Ratio of unique visitors who agreed to participate/unique first survey page visitors)		see above
Completion rate (Ratio of users who finished the survey/users who agreed to participate)		Informed consent had to be gathered via postal letter.
**Preventing multiple entries from the same individual**		
Cookies used		Cookies were not saved due to data protection regulations.<br>To identify participants a unique log in code was provided. However, once, the questionnaire was completed, participants could not access their questionnaire again.
IP check		IP addresses were not saved due to data protection regulations.
Log file analysis		No other techniques to analyze the log file for identification of multiple entries were used.

Item Category	Checklist Item	Methodological pilot study
Registration		Every potential participant received a unique log in code. This unique log in code was valid for the complete data gathering phase. As soon as the whole questionnaire was filled out, the respondent could not log in again. However, if a respondent interrupted answering, the system stored the number of pages and allowed to continue at the last filled out question.
**Analysis**		
Handling of incomplete questionnaires		Only completed online questionnaires were analyzed.
Questionnaires submitted with an atypical timestamp		No timeframe was set. However, the item-missingness was calculated and below 1% overall for the online questionnaire.
Statistical correction		We didn’t use weighting procedures, but we adjusted the analysis for socio-demographic characteristics

1. Eysenbach G. Improving the Quality of Web Surveys: The Checklist for Reporting Results of Internet E-Surveys (CHERRIES). Journal of Medical Internet Research. 2004 Jul-Sep;6(3):e34. PMID: PMC1550605. doi: 10.2196/jmir.6.3.e34.
