# Supplementary material for: Mode Equivalence of Health Indicators Between Data Collection Modes and Mixed-Mode Survey Designs in Population-Based Health Interview Surveys for Children and Adolescents: Methodological Study
Source: J Med Internet Res. 2018 Mar 5;20(3):e64. doi: 10.2196/jmir.7802 (PMC5859740; doi:10.2196/jmir.7802)
Supplement: Multimedia Appendix 2 [file jmir_v20i3e64_app2.pdf]

Appendix 1: Sociodemographic characteristics of responding parents of children aged 0–17 years by survey design and data collection mode

	Single-Mode Design	Mixed-Mode Design				All Designs		
		**sequential**	**concurrent**	**preselect**	***P-value (Chi^2)***	**SAQ-Web**	**SAQ-Paper**	***P-value (Chi^2)***
	n = 970	n = 854	n = 946	n = 698		n = 708	n = 2,760	
	%	%	%	%		%	%	
**Sex (child)**								
Male	47.5	52.1	47.6	51.3		52.5	48.6	
Female	52.5	47.9	52.4	48.7	.10	47.5	51.4	.06
**Age group (child)**								
≤ 2 years	14.3	15.1	15.3	12.9		15.3	14.3	
3–6 years	23.1	26.7	23.6	23.1		27.4	23.3	
7–10 years	21.3	24.1	24.5	22.1		26.1	22.2	
11–13 years	17.3	15.6	16.5	18.9		15.1	17.5	
**≥ 14 years**	23.9	18.5	20.1	23.1	.22	16.1	22.7	< .001
Yes<br>**Migration background (child)**	11.2	11.7	12.2	9.7	.43	11.6	11.2	.21
**Region of residence**								
West Germany	47.2	49.3	48.8	46.7		51.0	47.3	
East Germany/Berlin	52.8	50.7	51.2	53.3	.41	49.0	52.7	.08
**Municipality size**								
< 20,000	47.3	49.4	49.4	49.3		46.9	49.3	
20,000–99,999	22.8	20.7	21.8	19.6		19.6	21.8	
≥ 100,000	29.9	29.9	28.9	31.1	.85	33.5	28.9	.05
**Parental education** [a]								
Indeterminate	.3	.5	.3	0.0		0.0	.4	
Primary	7.7	7.7	8.5	6.4		4.1	8.6	
Secondary	56.6	57.3	57.7	52.1		49.9	57.8	
**Tertiary**	35.4	34.5	33.5	41.4	.07	46.0	33.3	<.001
Missing<br>**Net household income** [b]	10.6	10.5	11.0	9.0		4.8	11.8	
Low (1st quartile)	18.0	20.6	19.0	16.9		15.1	19.6	
Middle (2nd–4th quartile)	58.5	54.1	57.4	58.6		59.0	56.6	
High (5th quartile)	12.9	14.8	12.6	15.5	.11	21.0	11.9	< .001
**Participating parent**								
Missing	.4	.1	.4	.4		0.0	.4	
Mother	78.1	78.3	80.0	78.9		74.0	80.1	
Father	6.2	8.4	6.9	6.9		11.4	5.9	
Mother and father together	14.3	12.4	12.1	13.8		14.4	12.8	
Other	.9	.7	.6	0.0	.60	.1	.7	< .001
**Parental age**								
≤ 30 years	11.3	12.8	10.9	8.7		11.1	11.1	
31–40 years	42.4	44.5	44.8	44.1		45.3	43.6	

	Single-Mode Design	Mixed-Mode Design				All Designs		
		sequential	concurrent	preselect	*P-value (Chi²)*	SAQ-Web	SAQ-Paper	*P-value (Chi²)*
	n = 970	n = 854	n = 946	n = 698		n = 708	n = 2,760	
	%	%	%	%		%	%	
41–50 years	40.2	37.2	40.2	43.6		39.1	40.4	
> 50 years	6.0	5.5	4.1	3.6	.08	4.5	5.0	.84
**Parental marital status**								
69.470.170.874.669.3Missing	1.0	.9	.6	1.1		.1	1.1	
Separated/divorced/widowed71.1 Married or cohabiting	11.9	9.5	11.3	9.5		7.8	11.4	
Single	16.0	20.1	18.0	18.6	.31	17.5	18.2	.002
SAQ-Paper = Self-administered Paper-based Questionnaire; SAQ-Web = Self-administered Web-based Questionnaire								

[a] Education level assessed with the educational classification scheme “Comparative Analysis of Social Mobility in Industrial Nations” (CASMIN)
[b] Equivalent income according to the scale of the Organization for Economic Co-operation and Development (OECD); median according to the Statistics on Income and Living Conditions (EU-SILC) 2010
