# Supplementary material for: Mode Equivalence of Health Indicators Between Data Collection Modes and Mixed-Mode Survey Designs in Population-Based Health Interview Surveys for Children and Adolescents: Methodological Study
Source: J Med Internet Res. 2018 Mar 5;20(3):e64. doi: 10.2196/jmir.7802 (PMC5859740; doi:10.2196/jmir.7802)
Supplement: Multimedia Appendix 3 [file jmir_v20i3e64_app3.pdf]

Appendix 2: Sociodemographic characteristics of responding adolescents aged 11–17 years by survey design and data collection mode

	Single-Mode Design	Mixed-Mode Design				All Designs		
		**sequential**	**concurrent**	**preselect**	***P-value (Chi²)***	**SAQ-Web**	**SAQ-Paper**	***P-value (Chi²)***
	n = 344	n = 269	n = 290	n = 292		n = 299	n = 895	
	%	%	%	%		%	%	
**Sex (child)**								
Male	46.1	52.0	44.5	49.3		55.2	45.4	
Female	53.9	48.0	55.5	50.7	.17	44.8	54.6	.03
**Age group (child)**								
11–13 years	40.5	45.7	46.2	45.2		44.8	44.0	
≥ 14 years	59.5	54.3	53.8	54.8	.28	55.2	56.0	.56
**Migration background (child)**								
Yes	12.2	11.9	12.4	12.7	.94	13.7	11.8	.66
**Region of residence**								
West Germany	47.5	50.9	48.3	46.9		52.5	46.9	
East Germany/Berlin	52.5	49.1	51.7	53.1	.88	47.5<br>53.1	.10	
**Municipality size**								
< 20,000	47.8	53.5	46.9	52.1		50.8	49.6	
20,000–99,999	21.6	20.4	23.1	18.5		21.7	20.7	
≥ 100,000	30.6	26.0	30.0	29.5	.63	27.4	29.7	.74
**Parental education** [a]								
Indeterminate	.6	0.0	0.0	0.0		0.0	0.2	
Primary<br>8.4	7.1	10.0	9.5		1.6	10.8		
Secondary	58.4	63.4	60.5	50.5		56.6	58.4	
Tertiary	32.6	29.5	29.5	39.9	.08	41.8	30.5	<.001
**Net household income** [b]								
Missing	12.0	9.8	11.4	9.5		5.3	12.3	
Low (1st quartile)	16.2	18.8	18.1	17.6		14.3	18.4	
Middle (2nd–4th quartile)	56.6	57.6	59.8	53.5		56.1	57.0	
High (5th quartile)	15.3	13.8	10.7	19.4	.42	24.2	12.2	< .001
**Education attained or aspired to (child)**								
Unknown	4.4	9.3	4.8	5.5		8.4	5.0	
Low	5.0	4.5	5.2	4.5		2.7	5.5	
Middle	27.7	24.9	30.0	27.1	21.7	29.4		
High	63.0	61.3	60.0	63.0	.58	67.2	60.1	.002
SAQ-Paper = Self-administered Paper-based Questionnaire; SAQ-Web = Self-administered Web-based Questionnaire								

[a] Education level assessed with the educational classification scheme "Comparative Analysis of Social Mobility in Industrial Nations" (CASMIN)

[b] Equivalent income according to the scale of the Organization for Economic Co-operation and Development (OECD); median according to the Statistics on Income and Living Conditions (EU-SILC) 2010
